# Early Life Adversity as a Risk Factor for Visceral Pain in Later Life: Importance of Sex Differences

**DOI:** 10.3389/fnins.2013.00013

**Published:** 2013-02-13

**Authors:** Aaron Chaloner, Beverley Greenwood-Van Meerveld

**Affiliations:** ^1^Oklahoma Center for Neuroscience, University of Oklahoma Health Science CenterOklahoma City, OK, USA; ^2^Veterans Affairs Medical Center, University of Oklahoma Health Science CenterOklahoma City, OK, USA; ^3^Department of Physiology, University of Oklahoma Health Science CenterOklahoma City, OK, USA

**Keywords:** early life stress, neonatal stress, visceral pain, HPA, sex differences, glucocorticoid

## Abstract

A history of early life adversity (ELA) has health-related consequences that persist beyond the initial maltreatment and into adulthood. Childhood adversity is associated with abnormal glucocorticoid signaling within the hypothalamic-pituitary-adrenal (HPA) axis and the development of functional pain disorders such as the irritable bowel syndrome (IBS). IBS and many adult psychopathologies are more frequently diagnosed in women, and ovarian hormones have been shown to modulate pain sensitivity. Therefore, the sexually dimorphic effects of ELA and the role of ovarian hormones in visceral pain perception represent critical research concepts to enhance our understanding of the etiology of IBS. In this review, we discuss current animal models of ELA and the potential mechanisms through which ovarian hormones modulate the HPA axis to alter nociceptive signaling pathways and induce functionally relevant changes in pain behaviors following ELA.

## Introduction

Early life adversity (ELA) including parental neglect, chronic physical abuse, sexual abuse, and social stress profoundly affects brain development and increases the risk for adult psychopathologies. ELA is common with nearly 40% of Americans reporting exposure to a traumatic event prior to 13 years of age, and represents a substantial public health burden (Koenen et al., 2010). A positive correlation has been observed between ELA and comorbid pathophysiological, behavioral, and social problems in adulthood, suggesting that the effects of ELA are pervasive across a broad spectrum of adult disorders (Anda et al., 2006). Furthermore, childhood abuse increases the likelihood of adult gastrointestinal (GI) disorders such as irritable bowel syndrome (IBS), a prevalent functional GI disorder which affects 15–20% of the population and is characterized by chronic abdominal pain due to increased awareness of visceral stimuli and abnormal bowel habits (Whitehead et al., 1990; Talley et al., 1991). In a recent clinical study, IBS patients were two to four times more likely to report a history of ELA compared to healthy control subjects (Bradford et al., 2012). Importantly, psychosocial factors, such as a history of childhood abuse, are thought to elicit abnormal bidirectional communication along the brain-gut axis to influence IBS symptomatology (Ringel and Drossman, 2002; Camilleri, 2004). To date, the precise mechanisms by which ELA induces visceral hypersensitivity and abnormal pain reporting remain to be determined.

Dysregulation of the hypothalamic-pituitary-adrenal (HPA) axis is often observed in patients previously exposed to childhood abuse and is commonly reported in IBS patients (Dinan et al., 2006; Tarullo and Gunnar, 2006; Chang et al., 2009). Specifically, abnormal functioning of the amygdala, an important limbic structure involved in the facilitation of the HPA axis has been observed in IBS patients and in adults following childhood trauma (Bonaz et al., 2002; Naliboff et al., 2003; Wilder-Smith et al., 2004; Tottenham et al., 2010). Abnormal glucocorticoid (GR) receptor signaling throughout the HPA axis is observed following ELA and in IBS patients (Dinan et al., 2006; Wilkinson and Goodyer, 2011). Therefore, it is likely that aberrant functioning of the HPA axis contributes to IBS symptomatology in response to ELA.

Although ELA increases the risk of persistent disorders later in life for both men and women, evidence suggests that females are more susceptible to the effects of ELA and the development of adult pathologies (Hyman et al., 2008; Fisher et al., 2009; Xie et al., 2012). Female IBS patients were more likely to report ELA than healthy controls, whereas ELA history was similar between male IBS patients and controls, suggesting that women are more vulnerable to the enduring effects of ELA on the development of functional abdominal pain in adulthood (Bradford et al., 2012). Clinical data have shown that functional pain disorders including IBS, chronic pelvic pain, and painful bladder syndrome are more common in women, suggesting a sexually dimorphic pathophysiology of these disorders (Berkley, 1997; Lee et al., 2001). Although the precise mechanisms which contribute to the female predominance of these functional pain disorders are unknown, imaging studies revealed gender differences in regional brain responses to visceral stimuli, specifically showing greater amygdala activation following rectal distension in women compared to men (Berman et al., 2000). In addition to unique patterns of brain activation, the sex-related bias of these disorders, particularly following ELA, suggest that ovarian hormones play a prominent role in visceral hypersensitivity. Indeed, previous evidence illustrates that sex differences in pain sensitivity are influenced by cyclical changes in ovarian hormones (Hellstrom and Anderberg, 2003). Specifically, symptom exacerbation and increased rectal sensitivity are observed in female IBS patients during menses, and pain sensitivity and perceived stress are also increased during high cyclical levels of estrogen and progesterone in fibromyalgia patients (Heitkemper et al., 1995; Kane et al., 1998; Korszun et al., 2000; Houghton et al., 2002). These studies strongly support that circulating ovarian hormones modulate pain behavior; however the precise mechanisms have yet to be elucidated.

Due to the difficulty of controlling for confounding factors within the clinical population and ethical limitations, a systematic investigation of the mechanisms responsible for enhanced adult pathologies following childhood adversity is very difficult in human subjects. Therefore, rodent models of ELA have provided considerable insight into the persistent effects of early life maltreatment on pain and behavior (Barreau et al., 2007). Although sex-related biases have been reported clinically, rodent models of adult psychopathologies and pain disorders are predominantly performed in male rats. This is particularly true when investigating the long-term impact of ELA on adult behavior and working exclusively with males allows for a less complex experimental design and fewer complications with animal housing. Therefore, very little is known about the role of ovarian hormones in these adult syndromes, specifically in the context of ELA.

This review summarizes the recent literature on the effect of ELA on abnormal pain perception with a particular focus on sex differences. In this review, we discuss the potential mechanisms of increased pain following ELA and focus on the role of ovarian hormones and HPA-mediated mechanisms. In addition, we discuss the central processes involved in persistent abnormalities of pain perception and anxiety following ELA and highlight specific structures involved in the pain perception including the hippocampus and amygdala. Together, this review provides an important summary of important findings and suggests potential areas that may represent useful therapeutic targets for the treatment of pain disorders, such as IBS following ELA.

## Relevant Animal Models of ELA

The most documented model of ELA is maternal separation (MS), which consists of removing pups from the nest, mother, and home cage for varying periods of time during the early postnatal period (PN2–14; Plotsky and Meaney, 1993). MS has differential effects in adulthood that depend on the duration of separation and the specific number of separation days, which largely appear due to altered maternal behavior upon the pups return to the nest. For instance, a brief separation of 15 min per day from PN2 to PN14 results in increased maternal attention upon return of the litter (heightened licking and grooming and increased arched-back nursing) that causes decreased reactivity of the HPA axis in response to stress of the offspring in adulthood (Levine, 1967; Plotsky and Meaney, 1993). However, more substantial MS (3 h from PN2 to PN14) decreased licking and grooming and arched-back nursing (LG-ABN) by the mother and caused hyper-reactivity of the HPA axis in adulthood. In addition to the changes in HPA activation, differential effects on visceral sensitivity are observed depending on the MS paradigm. Separation for 3-h per day induced visceral hypersensitivity quantified as an exaggerated visceromotor response (VMR) to colorectal distension (CRD) and at baseline and following acute stress as compared to non-handled and handled (15 min MS) controls (Coutinho et al., 2002). Furthermore, MS reduces opioid-mediated nociceptive inhibition of visceral pain responses in adulthood (Coutinho et al., 2002). Therefore, MS represents a simple and easily reproducible model of ELA that mimics the clinical situation of altered HPA activity and visceral hypersensitivity in adulthood.

Another model of ELA that is becoming more widely used is the model of limiting the available nesting material (Gilles et al., 1996). In this model, rat mothers and pups are housed in a wire-bottom cage and given a single sheet of paper towel as nesting material from PN2 to PN9. The limited nesting model induces sporadic, fragmented, and unpredictable maternal behavior during the ELA period (Ivy et al., 2008). These maternal behaviors are consistent with those observed in the MS paradigm; however the limited nesting model has the unique advantage of inducing abnormal maternal care without separating the dam from her pups. In the clinical situation of neglect and abuse, the mother is generally present but exhibits improper and abnormal care (Whipple and Webster-Stratton, 1991; Kendall-Tackett, 2007). Therefore, limited nesting is a clinically relevant model of ELA, which has been shown to affect the HPA-responsiveness of pups and alter nociceptive sensitivity in adulthood (Avishai-Eliner et al., 2001; Green et al., 2011).

A third model of ELA relies on neonatal colonic irritation (nCI) via either colonic infusion of mustard oil or repeated CRD during the early neonatal period (Al-Chaer et al., 2000). Although this model of ELA relies on colonic manipulation, no overt or histological changes are observed to the colonic mucosa in adulthood. nCI has been shown to cause visceral hypersensitivity characterized by increased neuronal excitability along with changes in ionic conductance across the colonic mucosa in adulthood (Al-Chaer et al., 2000; Lin and Al-Chaer, 2003, 2005; Chaloner et al., 2010). Neonatal pain has been shown to induce neurogenesis; therefore, visceral hypersensitivity following nCI may result from enhanced neurogenesis directly in primary pain processing areas (Leslie et al., 2011). Furthermore, nCI increases spinal neuronal excitability and induces thoracolumbar and lumbosacral visceral afferent sensitization (Al-Chaer et al., 2000; Lin and Al-Chaer, 2003).

Models of ELA often mimic the clinical situation of psychological trauma and neglect, while ignoring ELA resulting from abusive relationships, which is an important contributor to the development of adult pathologies such as IBS (Bryer et al., 1987; Bradford et al., 2012). In addition, previous studies suggest that classical conditioning in response to previous experience with painful stimuli may contribute to increased pain sensitivity, particularly in IBS patients (Lembo et al., 1999; Nozu et al., 2006). Additionally, passage of hard stool in infancy causes intense pain and conditions the child to avoid defecation, which may contribute the development of altered bowel habits and chronic abdominal pain in adulthood (Al-Chaer and Hyman, 2007). Therefore, a conditioning model of ELA is consistent with human circumstances related to heightened pain and anxiety in adulthood; however most models of ELA lack a conditioning component. The animal model of ELA used in our laboratory utilizes neonatal Pavlovian conditioning and is particularly relevant to the understanding the mechanisms of IBS. This model, which has been well developed and characterized by Sullivan et al. (2000) relies on conditioned responses to an odor stimulus, and closely mimics the human situation of attachment to an abusive caregiver (Haroutunian and Campbell, 1979; Camp and Rudy, 1988). A conditioning model of ELA has an additional advantage that allows for control of shock predictability based on the presence or absence of an odor cue (Tyler et al., 2007). In this paradigm, paired odor-shock serves as a model of predictable adversity, whereas unpaired odor-shock is a model for unpredictable ELA. Using this model of ELA, we have shown that only unpaired (or unpredictable) infant experiences result in the development of visceral hypersensitivity and abdominal pain in adult rats as demonstrated by an increase in the number of abdominal contractions in response to nociceptive CRD (Tyler et al., 2007). This indicates that the predictability of ELA is important such that only unpredictable neonatal experiences will result in the development of visceral pain in adulthood. Additionally, through measurements in a light-dark box, which utilizes a rat’s avoidance of bright spaces compared to dark areas to assess anxiety, we have shown that animals receiving unpredictable ELA demonstrate an increase in anxiety as compared to paired and odor-only animals in adulthood (Tyler et al., 2007). These data suggest that a neonatal conditioning model of ELA accurately reproduces visceral hypersensitivity and altered anxiety-like behavior, which is consistent with the clinical setting of IBS (Whitehead et al., 1990, 2002).

## Role of Ovarian Hormones in Pain and Behavior

Sex differences in nociception have been reported clinically and observed in rodent models, and sex differences in regional brain activation in response to CRD are similar between rodents and human studies (Ji et al., 2006; Wang et al., 2009). Cyclical changes in pain sensitivity have been detected across the menstrual cycle and have also been observed in the rodent estrus cycle, with the greatest visceral sensitivity to luminal distension observed during proestrus, a phase characterized by heightened estradiol and progesterone (Sapsed-Byrne et al., 1996; Ji et al., 2008). Previous studies in rodent models have shown that females are more susceptible to developing visceral hypersensitivity following exposure to MS or odor-shock conditioning (Rosztoczy et al., 2003; Chaloner and Greenwood-Van Meerveld, 2013). These data provide support that ovarian hormones play a prominent role in maintaining the persistent effects of ELA on increased pain sensitivity in humans and rodent models. However, the mechanisms by which ovarian hormones modulate visceral sensitivity are poorly understood.

Ovarian hormones are important modulators of brain plasticity and organization (Fitch and Denenberg, 1998). The effects of ovarian hormones on physiological functions can be classified according to two broad categories: organizational and activational (McCarthy, 2008). The organizational effects of sex hormones generally occur during development and are established following initial exposure in the perinatal period; however evidence also suggests that central organizational effects may occur during puberty or late adulthood, effectively establishing the tone for the future fluctuations of ovarian hormones (Rodriguez-Sierra, 1986; McCarthy, 2008). The activational effects occur when sex hormones are present and readily available to act on their respective targets. These activational effects are thought to be mediated via the neuronal circuits that were established during the perinatal organizational period. However, the relative role of organizational vs. activational effects of ovarian hormones on adult pathologies following ELA has not been adequately studied. Data from our laboratory have shown that effectively abolishing the activational role of ovarian hormones via ovariectomy (OVX) reverses the effects of ELA on adult visceral pain hypersensitivity in female rats (Chaloner and Greenwood-Van Meerveld, 2013). Furthermore, reintroduction of ovarian hormones via a subcutaneous estradiol pellet was sufficient to induce visceral hyperalgesia to nociceptive CRD (Chaloner and Greenwood-Van Meerveld, 2013). Clinical evidence confirms that the activational effects of ovarian hormones play a direct role in visceral pain modulation. Previously, Mathias et al. (1994a,b) demonstrated that pharmacological OVX via leuprolide acetate treatment dramatically diminishes IBS-like symptomatology in female. In addition, pain sensitivity is reduced in female IBS patients following menopause (Palsson et al., 2003), showing a direct role for the activational effects of ovarian hormones in modulating visceral pain. The effects of hormonal changes during pregnancy on visceral pain have been difficult to quantify due to the similarity in frequently reported complaints of pregnancy and IBS symptomatology (i.e., nausea, abdominal pain, constipation, and diarrhea), and require further investigation (Bruno, 2004).

An important ovarian hormone that directly modulates pain perception in clinical studies and animal models is estradiol or estrogen. Estrogens can act as neurosteroids to increase glutamate-mediated excitation, reduce GABAergic inhibition, increase hippocampal c-Fos expression, and potentiate synaptic plasticity, effectively increasing neuronal excitability throughout the central nervous system (CNS; Aloisi and Bonifazi, 2006). This increased neuronal excitability may drive heightened nociception in females, which is observed during periods of high estrogens. Indeed, fibromyalgia symptomatology is highest in women when estrogen is highest in the luteal phase of the menstrual cycle (Korszun et al., 2000). Estradiol and progesterone have been shown to directly modulate somatic pain behavior in rats (Kayser et al., 1996). In addition, estradiol implanted directly onto the central nucleus of the amygdala (CeA) was shown to increase visceral sensitivity to CRD in a rodent model (Myers et al., 2011). The role of estradiol in nociception can also be observed in males. For instance, intracerebroventricular (i.c.v.) estradiol has been shown to increase licking following subcutaneous formalin injection in male rats, and estradiol localized to the amygdala increased visceral pain behavior in males (Aloisi and Ceccarelli, 2000; Myers et al., 2011). These data highlight the importance of estradiol in nociception and pain behavior.

## ELA and the Stress Axis

During early neonatal development rat pups exhibit low basal levels of the stress hormone corticosterone (CORT; cortisol in humans), and reduced activation of the HPA axis in response to stress. This stage of reduced HPA activation, which occurs during the first 2 weeks of life in the rat, is known as the stress hyporesponsive period (SHRP) and represents a critical time for the development of a normal stress axis (Meaney and Aitken, 1985; De Kloet et al., 1988). In rodents, the SHRP is maintained by appropriate interactions in the mother-pup relationship, which relies heavily on arched-backed nursing and appropriate licking and grooming behaviors (Levine, 1994; Aisa et al., 2008). Quiescence of the HPA axis during the SHRP is crucial for the development of a normal stress response. Disruptions in mother-pup interactions, as in the limited nesting or MS models, lead to the development of a premature adult-like stress response, and potentially cause permanent changes in adult HPA functionality (Plotsky et al., 2005). Normally, stress causes the release of corticotrophin-releasing factor (CRF) from the parvocellular cells of the paraventricular nucleus of the hypothalamus (PVN) into the hypophyseal portal system. CRF then acts on the anterior pituitary gland to signal the release of adrenocorticotrophic hormone (ACTH), which enters systemic circulation to interact with the adrenal cortex and ultimately results in the release of CORT. CORT then acts on the hippocampus, PVN, and pituitary to activate a negative feedback mechanism and shut down further HPA activation (De Kloet and Reul, 1987; Ladd et al., 2004). In the brain, CORT mediates its actions via GR and mineralocorticoid receptors (MR), which have distinct distribution and function throughout the CNS. Receptor binding studies indicate that MR has a 10-fold greater affinity for CORT than GR; therefore, when CORT is low, such as at basal levels, the observed effects are mediated through MR. Under stressful conditions, MR becomes saturated, and CORT is free to act on GR to induce the negative feedback mechanisms of the HPA axis (Francis et al., 1999; Weaver et al., 2004). Although little is known about the effects of ELA on MR, ELA has been shown to significantly alter GR expression and the functioning of the HPA axis in clinical studies and animal models. For example, prolonged MS increases CRF mRNA density in the PVN and decreases GR expression in the hippocampus (Ladd et al., 2004; Aisa et al., 2008). Reduced hippocampal GR may contribute to allostatic overload or a deficiency in the negative feedback mechanisms of the HPA axis which allows for hyperactivation in adulthood. Indeed, exposure to ELA results in an overactive HPA axis with increased ACTH peak and prolonged elevated CORT levels in response to air puff startle (Plotsky et al., 2005). Meaney and colleagues have further advanced these observations in a model which utilizes naturally occurring variations in maternal behavior to separate “good moms” and “bad moms” based on high vs. low LG-ABN, respectively. Their results have provided intriguing data on reduced negative feedback mechanisms in the HPA axis following poor maternal care. In their studies, low LG-ABN mothers produce offspring with reduced expression of hippocampal GR (Liu et al., 1997; Francis et al., 1999). Through the use of cross-fostering, decreased hippocampal GR has been shown to be an effect of environment, rather than due to heritable changes in behavior (Francis et al., 1999). Furthermore, the reduction in GR in low LG-ABN offspring is maintained into adulthood as a direct result of epigenetic modification through enhanced methylation of exon 1_7_, the GR promoter region, which inhibits binding of nerve growth factor-inducible protein A, a transcriptional activator GR (Weaver et al., 2004). These data provide support that subtle changes in maternal behavior, whether naturally occurring or induced via MS or limited nesting, can alter the functioning of the HPA axis and result in reduced negative feedback of the system, contributing to stress-related pathologies in adulthood.

## Ovarian Hormones and the HPA Axis

Women are more vulnerable to develop anxiety disorders following traumatic events, suggesting a role for ovarian hormones in influencing the HPA axis activation (Yonkers and Ellison, 1996; Seeman, 1997; Isgor et al., 2003). In rodent models, females exhibit greater ACTH and CORT release in response to stress as compared to males, and a direct correlation between ovarian hormones and HPA activation has been shown where plasma and adrenal CORT levels are the highest during the proestrus phase, a period of the estrous cycle characterized by elevated serum estradiol (Raps et al., 1971; Handa et al., 1994). These data suggest that high levels of estradiol and progesterone increase activation of the HPA axis. A direct relationship between estradiol and HPA activation has been shown in a recent study where i.c.v. administration and infusion of estradiol to the PVN caused a significant increase in CORT, showing that central estradiol has profound effects on the HPA axis (Liu et al., 2012). The actions of estradiol or estrogen within the brain are mediated via two types of receptors, estrogen receptor alpha (ERα) and ERβ, which have distinct distribution and functions within the CNS. Previous results have implicated an important role of ERα in the regulation of the HPA axis. Estradiol and propylpyrazole triol, an ERα agonist, significantly increased the CORT response to a 30 min restraint stressor when directly infused onto the PVN, whereas the ER antagonist ICI 183,780 reduced stress-induced CORT release (Liu et al., 2012). ERα agonist treatment in OVX rats increased CORT and ACTH levels in response to stress, increased activation of the PVN, and impaired dexamethasone-induced suppression of CORT release (Weiser and Handa, 2009). These data suggest that estradiol, acting through central ERα, reduces negative feedback mechanisms of the HPA axis, similar to what was previously described following ELA. Furthermore, estradiol may mediate its effects on HPA activation through transcriptional regulation of specific gene targets, which are important mediators of the HPA axis (Kageyama and Suda, 2009). Estrogen response elements (EREs) have been observed in the CRF gene promoter region and estradiol and diarylpropionitrile (DPN), an ERβ selective agonist, directly increased CRF expression in hypothalamic 4B cells (Vamvakopoulos and Chrousos, 1993; Ogura et al., 2008). In addition, high estradiol replacement increased basal hypothalamic CRF and elevated plasma CORT and ACTH in OVX rats, which provides functionally relevant evidence that estradiol regulates CRF expression *in vivo* (Ochedalski et al., 2007). Estradiol-mediated regulation of GR transcription has also been found in breast cancer cell lines. These studies have shown that estradiol treatment significantly reduces GR expression and increases protein phosphatase 5 activity, which dephosphorylates and subsequently inactivates GR (Krishnan et al., 2001; Zhang et al., 2009). Additionally, ER activation has been shown to target GR for proteosomal degradation and reduce GR protein expression (Kinyamu and Archer, 2003). The combined action of estradiol through ERα-mediated action, direct transcriptional alterations, and post-translational regulation reduces the negative feedback of the HPA axis by decreasing GR and increasing CRF-mediated signaling.

## Evidence to Support Abnormalities in Amygdala Activity in Response to ELA

In addition to the changes observed in the hippocampus, previous studies suggest that ELA alters other key brain areas involved in programming of the HPA axis including the amygdala and the locus coeruleus (LC; Liu et al., 1997; Caldji et al., 1998; Sevelinges et al., 2007; McGowan et al., 2009). While the effect of elevated CORT in the brain and periphery is inhibitory as a component of the negative feedback on the HPA axis within the PVN and hippocampus, CORT acting at the level of the amygdala serves to facilitate the HPA axis. Exposure to ELA causes abnormal amygdala development and increases CRF expression within the amygdala, which may cause persistent abnormalities and enhanced facilitation of the HPA axis (Cratty et al., 1995; Hatalski et al., 1998; Vazquez et al., 2006; Becker et al., 2007). Additional studies using MS have further characterized this altered expression by showing sexually dimorphic changes in CRF immunoreactivity within the CeA following ELA (Desbonnet et al., 2008). Furthermore, exposure to adverse care from infancy to early and late childhood significantly increases amygdala volume and enhances amygdala activity in the clinical population (Mehta et al., 2009; Tottenham et al., 2010). Although the mechanism by which ELA alters amygdala activity is unclear, data suggest that aberrant amygdala functioning following ELA results in enhanced CRF release to facilitate the stress response, which may contribute to increased anxiety (Hatalski et al., 1998; Becker et al., 2007). The amygdala has also been shown to be an important structure involved in the modulation of pain, and may be particularly relevant to enhanced visceral pain in IBS (Berman et al., 2000; Naliboff et al., 2003; Neugebauer et al., 2004). Data from our laboratory have highlighted the importance of CORT acting at the level of the amygdala to increase visceral pain behavior in a rodent model (Greenwood-Van Meerveld et al., 2001; Myers and Greenwood-Van Meerveld, 2007, 2010, 2012). The precise mechanism by which amygdala activation induces heightened visceral sensitivity is unknown; however, the amygdala has diffuse connections to important brain nuclei involved in pain modulation including the hypothalamus, LC, Barrington’s nucleus, dorsal motor nucleus of the vagus, nucleus of the solitary tract, the raphe nucleus, periaqueductal gray, and the parabrachial nucleus (Myers and Greenwood-Van Meerveld, 2009). Therefore, hyperactivation of the amygdala may serve to increase not only HPA activity but also contribute to enhanced visceral pain perception following ELA.

## Concluding Remarks

Visceral pain is the hallmark feature of IBS, a prominent GI disorder with a substantial socioeconomic burden of approximately $1.7 billion a year in direct costs. However the etiology and mechanisms of IBS are poorly understood and efficient therapeutic interventions have yet to be developed (Hulisz, 2004). A relationship between ELA and the development of IBS in adulthood has been described in clinical literature and animal models. Furthermore, both ELA and IBS are characterized by female predominance and dysregulation of the HPA axis (Drossman et al., 1997; Whitehead et al., 2002; Dinan et al., 2006; Tarullo and Gunnar, 2006; Hyman et al., 2008; Chang et al., 2009). Therefore, the mechanisms by which ELA induces visceral hypersensitivity likely involve ovarian hormones and signaling within the HPA axis. In this review, we discussed previous research in rodent models that demonstrated increased visceral pain behaviors following ELA and the advantages and disadvantages of relevant models of ELA. Data from rodent literature suggests that ELA directly influences nociception and alters signaling in the HPA axis, with specific changes in amygdala-mediated mechanisms. Therefore, it is our hypothesis that ELA sets the stage to increase pain sensitivity and enhance the responsiveness of the HPA axis, either through reduced negative feedback or increased facilitation. Following maturity, the activational effects of ovarian hormones act to further increase this priming and ultimately contribute to the development of adult disorders such as IBS, fibromyalgia, and heightened anxiety. Therefore, ELA may serve as a predisposing factor, which can be further potentiated due to the effects of ovarian hormones to increase activation of pain pathways and the HPA axis, explaining the increased incidence of these disorders in women.

The importance of sex hormones and HPA signaling through CRF and GRs, particularly following ELA, may have a critical influence on the understanding of the pathophysiology and mechanisms of IBS. The data highlighted within this review strongly suggest that ovarian hormones, specifically estradiol, may be important modulators of the effects of ELA on visceral pain perception and HPA activation. Understanding these sexually dimorphic mechanisms of disease may lead to the development of more targeted and specific therapies which could have profound effects on management of visceral pain as seen in functional disorders such as IBS, where a clear etiology has not yet been identified.

## Conflict of Interest Statement

The authors declare that the research was conducted in the absence of any commercial or financial relationships that could be construed as a potential conflict of interest.
